# ‘I’ve just heard that there are people who feel like they need to exercise’: a photo-elicitation study of values and priorities influencing physical activity in a socioeconomically disadvantaged neighbourhood in Uppsala, Sweden

**DOI:** 10.1136/bmjopen-2024-085356

**Published:** 2024-08-29

**Authors:** Louise Engelbrektsson, Hedda Ottesen, Meena Daivadanam, Laran Matta, Helle Mølsted Alvesson

**Affiliations:** 1 Department of Global Public Health, Karolinska Institutet, Stockholm, Sweden; 2 Global Health and Migration Unit, Department of Women’s and Children’s Health, Uppsala University, Uppsala, Sweden

**Keywords:** qualitative research, cardiovascular disease, percieved social support, health policy, public health

## Abstract

**Abstract:**

**Objective:**

People living in socioeconomically disadvantaged neighbourhoods in Sweden engage less in physical activity compared with the general population, contributing to an elevated risk of cardiometabolic diseases. To inform targeted and effective public health interventions, understanding residents’ lived experiences is essential. This study sought to understand the values and priorities associated with physical activity by people living in a socioeconomically disadvantaged neighbourhood in Region Uppsala, Sweden, informing a public health intervention to prevent cardiometabolic diseases and promote healthy and active living.

**Design:**

The study employed a photo-elicitation methodology, combining participants’ photographs with semistructured interviews. Data were analysed using reflexive thematic analysis.

**Setting:**

A socioeconomically disadvantaged neighbourhood in the city of Uppsala, Sweden, characterised by a large proportion of households with low income, a large percentage of individuals living on economic aid, high unemployment rates, low educational attainment and high levels of poor health.

**Participants:**

A total of 15 participants (8 women and 7 men) were purposively sampled between February and August 2023 and recruited via fieldwork, social media and local stakeholders.

**Results:**

Participants described challenging conditions for physical activity, including conflicting values and priorities between themselves and the local authorities. Four main areas emerged as sources of tension; difficulties influencing decision-making processes affecting the neighbourhood, unmet needs of gender-separated physical activity spaces, discrepancy between the perceived pressure and individual motivation to be active, and their perception of health being solely an individualised responsibility, but their need of support.

**Conclusions:**

This study underscores the importance of understanding and navigating the values and priorities influencing physical activity among residents in a socioeconomically disadvantaged neighbourhood, when designing public health interventions. Findings reveal that residents’ needs for being physically active are not met by the authorities who are perceived to have different priorities, and that the lack of influence of citizen voices undermines trust in the local authorities.

**STRENGTHS AND LIMITATIONS OF THIS STUDY**

- A strength of this study is the use of the photo-elicitation methodology since the photographs prompted discussions and uncovered the meanings attached to the places, activities and facilities that were photographed.
- Furthermore, diverse participant demographics enriched the study’s findings and contributed to a comprehensive understanding of values and priorities in the context of super-diverse neighbourhoods in Sweden.
- However, the exclusion of individuals without proficiency in Swedish, due to language constraints, may have introduced a selection bias.

## Introduction

 Physical inactivity is estimated to cause 5.3 million premature deaths globally each year.^1^ It is a leading risk factor for non-communicable diseases such as cardiometabolic disease, including type 2 diabetes (T2D).^2^ Regular physical activity is recognised as a crucial factor in preventing or delaying the onset of cardiometabolic disease.^3 4^ However, the socioeconomic disparities in the performance of physical activity are large. In Sweden, the risk of developing T2D is three times higher in socioeconomically disadvantaged groups than in the general population.^5 6^

As in many other countries, promoting physical activity continues to be a priority for public health initiatives in Sweden. Nevertheless, from an equity standpoint, public health interventions often fail to target socioeconomically disadvantaged groups, as they are often developed based on the needs of the general population.^7^ In Sweden, socioeconomically disadvantaged neighbourhoods are heterogeneous, but all are characterised by high levels of poverty, high unemployment and low educational attainment.^8^ Socioeconomically disadvantaged neighbourhoods in Sweden and other parts of Europe have been shown to be characterised by superdiversity. That they encompass a wide range of ethnicities, religions, and educational and economic backgrounds, as well as time spent in the neighbourhood, further complicates the design and implementation of effective public health strategies.^9^ In addition, the common labelling of these neighbourhoods as ‘at risk’ or ‘socially vulnerable’ contributes to negative perceptions of the residents, fostering a sense of ‘outsideness’ in Swedish society. In turn, this undermines residents’ sense of identity, social position and political influence.^10^

Interventions lacking consideration for the unique needs and obstacles faced by socioeconomically disadvantaged groups may yield limited effectiveness.^7 11^ Failing to tailor public health interventions to the specific setting and population may, therefore, exacerbate health inequities. Consequently, it is crucial to understand the specific needs of socioeconomically disadvantaged groups to design successful physical activity interventions.^11 12^

Community-based strategies and codesign approaches have emerged as methodologies for developing more inclusive and culturally relevant interventions in socioeconomically disadvantaged settings in Sweden.^13 14^ These approaches emphasise active engagement with communities, prioritising their input and participation in the design, implementation and evaluation of health promotion initiatives. Community-based programmes have demonstrated promising results in fostering social cohesion, empowerment and collective action within disadvantaged communities, leading to improved health outcomes and reduced health inequities.^10 15 16^

Several studies have explored the barriers and facilitators associated with physical activity in disadvantaged neighbourhoods.^17^,^21^ Common barriers include lack of time, perceived lack of safety, perceived poor weather conditions, health issues, economic constraints and perceived lack of physical activity facilities.^1820^,^22^ Facilitators encompass aspects such as social support, social cohesion, strong leaders in the community and feelings of empowerment.^23^,^25^ However, while barriers and facilitators provide helpful insights, there is a need to further explore residents’ values and priorities in order to optimally tailor the design of a physical activity intervention, as these play a crucial role in commitment to health behaviour change.^26^

This study aimed to understand the values and priorities associated with physical activity of individuals living in a socioeconomically disadvantaged neighbourhood in Region Uppsala, Sweden. The results of this study will inform the design of a public health intervention aimed at preventing cardiometabolic diseases in Region Uppsala.

## Methods

### Design

This study was part of the formative studies of the PREVENT research project, which aims to codesign a community outreach component through patient and public involvement, to prevent cardiometabolic diseases in socioeconomically disadvantaged neighbourhoods in Region Uppsala, Sweden.^27^)

A qualitative study employing a photo-elicitation methodology was carried out. Photo elicitation is a visual and reflective participatory research approach where participants’ photos are paired with in-depth interviews.^28 29^ The methodology, with its participant-driven structure, increases the level of participants’ involvement in the research process.^26^ By incorporating participant-generated photographs, the study aimed to obtain a rich and visual representation of the participants’ lived environments, uncovering valuable insights into their perceptions and experiences related to physical activity.^28^ The interpretive engagement framework was used to facilitate the process, supporting a joint analysis of photographs and interviews.^30^ The framework supported participant reflexivity, the interpretation of the researcher, the theoretical basis of the study and research field, as well as new knowledge claims. The study adopts a qualitative approach within a social constructivist paradigm, viewing knowledge as being developed through the experiences and perspectives of the participants.

The Consolidated Criteria for Reporting Qualitative Research guidelines were used to report this study^31^ (online supplemental appendix 1).

### Setting

The study was conducted in a socioeconomically disadvantaged neighbourhood in Region Uppsala. Compared with other neighbourhoods in the region, it is characterised by a high share of households with low income, a large share of individuals living on economic aid, high unemployment rates, low educational attainment and high rates of poor health.^8^ The neighbourhood’s Care Need Index, which measures the level of social deprivation, is twice as high as the regional neighbourhood average.^8 32^ The neighbourhood is dominated by residential buildings, with apartment complexes closer to the centre, and individual houses located on the periphery. There are several parks of different sizes in the neighbourhood as well as a number of playgrounds. Where the residential area ends, an extensive green area takes over with a large, open lawn and a forest with lighted paths. There are walking paths and bike lanes in the central and peripheral parts of the residential area and in the forest close by. In the central part of the neighbourhood, there is a church, a municipality area office and a large shopping mall with grocery stores, clothing stores, hairdressers, a primary healthcare centre, a library and a few non-governmental organisations.

### Patient and public involvement

Citizen engagement is a form of patient and public involvement that was employed in this study.^33^ This study is formative and is the first step of a long process of citizen engagement to inform a codesigned public health intervention with community outreach, aimed at promoting healthy and active living in the community. During the recruitment of study participants, the first author shared information about the study on social media platforms and was often present in the community, engaging in different community activities, such as women’s cafes, an open preschool, the church cafe and open activities at local organisations. This allowed the residents to easily contact her if they wished to participate in the study. The participants chose where and when they wanted to meet up for the interview.

### Recruitment and consent

A total of 15 participants were recruited using purposive sampling through fieldwork, social media platforms and local stakeholders. We aimed to achieve a diverse representation across several sociodemographic variables, including gender, age, language backgrounds, income levels, educational backgrounds and physical activity behaviour. Specifically, we sought to include participants from the same demographic groups that the intervention would target. Participants were screened using the Finnish Diabetes Risk Score (FINDRISC) before being enrolled in the study, to stratify their risk of diabetes. Eligible participants were between 25 and 75 years old and resided in the neighbourhood. Exclusion criteria included already being diagnosed with T2D, suffering from any chronic or acute disease that would limit the ability of the person to participate in the study, and inability to provide informed consent. If the participant had a high FINDRISC score (15 or above) he or she was advised to contact the healthcare centre. There was no participant drop-out in this study and all participants provided written and oral informed consent. The participants’ characteristics are presented in table 1.

**Table 1:** Characteristics of participants interviewed

Characteristics		Participants (n=15)
Age (md)		41 (30–72)
Gender		
	Women	8
	Men	7
Country of birth		
	Sweden	7
	Germany	1
	UK	1
	Burundi	1
	Eritrea	1
	Turkey	2
	Syria	2
Occupational status		
	Employed	6
	Self-employed	4
	Retired	3
	Sick leave	2
Years resided in the neighbourhood		
	1–3 years	5
	4–6 years	3
	7–9 years	2
	10+ years	5
FINDRISC		
	Low risk	9
	Moderate risk	3
	High risk	3

FINDRISCFinnish Diabetes Risk Score

### Data collection

Data collection was undertaken by the first author, a female researcher with a master’s degree in public health and took place between February and August 2023. The research team was composed of researchers in the field of global health and medical anthropology, with extensive expertise in their fields. Furthermore, two of the coauthors are medical doctors.

The first author met with all interested participants to introduce the study and go through the consent form and guidelines for the photographs to be taken. Participants were encouraged to take about 10 pictures of places or activities in their everyday lives that facilitated or hindered their physical activity. The participants used their own mobile phones to take the pictures.

After a 7-day period, the first author and the participant met again for a semistructured interview, guided by the photos taken by the participant and facilitated by an interview guide (online supplemental file 1). The interview guide comprised seven primary questions, focusing on the participants’ photos and perceptions of physical activity within their residential area. Since only minor revisions were made following the pilot interview, the first participant was included in the study. For each of the photos, participants described the place or activity depicted, as well as their experiences and feelings related to the photo. Follow-up questions and probes were used during the interviews to secure an in-depth understanding of the participants’ perceptions and experiences. To gain insights into the participants’ priorities and values, the questions focused on meanings and experiences attached to the particular place or activity.

The interviews were carried out at a place suggested by the participant and lasted between 37 min and 1 hour and 6 min. The number of photographs taken ranged from 7 to 32. All interviews were conducted in Swedish and all participants were able to speak the language confidently. The interviews were conducted by the first author and were recorded using a dictaphone. At the request of the participants, two interviews were carried out without photographs, using an adapted version of the interview guide. Data collection proceeded until the authors were satisfied that they had gathered sufficiently diverse and comprehensive information to meet the aim of the study. This was guided by the concept of information power, as described by Malterud.^34^

### Data analysis

The interviews were transcribed verbatim and coded inductively in NVivo V.1.7.1 software. In the analysis, a reflexive thematic approach, as suggested by Braun and Clarke, was employed.^35^) The iterative process involved revisiting the data to assess codes and eventually develop themes. Codes were compared with find patterns and categories, and subthemes and themes were then identified. The coding process involved four researchers. Initially, the first author conducted the coding independently. The codes were then reviewed and discussed in a reflexive iterative process primarily with HO and HMA, followed by MD, to explore alternative interpretations of data throughout the process.

The visual data were analysed together with the interview transcripts to provide a more nuanced understanding of participants’ experiences.^30^ Interviews conducted during different seasons of the year were compared with assess whether differences in the results could be attributed to changes in weather conditions. To capture the nuances, the analysis was conducted in Swedish and codes were translated into English in the final stages of the analytical process. Member checking was not conducted due to feasibility issues, as participants were either unable or unwilling to be contacted further. Instead, preliminary results were presented at workshops with local stakeholders to facilitate discussions about conditions for physical activity. 

## Results

Findings are presented under the main theme: *They don’t understand our priorities, but it is my own fault not being more physically active*. This main theme comprises two subthemes: *We do not always feel like we have a say* and *I need more support but without any pressure*. The relationships between the main theme, subthemes and respective categories are presented in table 2. Quotations are followed by the participant’s pseudonym, gender, age and number of years residing in the neighbourhood. The number of years residing in the neighbourhood has been included since it was described by the participants to mark the start of a new chapter in their life where housing affordability started to play a significant role, for example following a major change in their life such as divorce.

**Table 2:** Subcategories, categories, subthemes and main theme

Subcategory	Category	Subtheme	Main theme
Negative perceptions and expectations of the neighbourhood are leading to a sense of collective punishmentThey don’t understand us, it is us against themThey are destroying our nature	Different priorities between the authorities and the residents	We do not always feel like we have a say	They don’t understand our priorities, but it is my own fault not being more physically active
Dialogue meetings fostering feelings of being neglectedThe need of gender separated exercise spaces is being ignored	Not feeling heard by the ones in power		
People are lonely here and there is nothing to doI don’t want to feel forced to show up to activities	Balancing support and autonomy	I need more support but without any pressure	
It is my own fault being physically inactiveI am not sure if I want to be more active	Personal responsibility vs. willingness to be physically active		

### They do not understand our priorities, but it is my own fault not being more physically active

The main theme highlights how the participants expressed a conflict in priorities between themselves and those with the perceived power regarding physical activity. Most participants identified the municipality as the actor having the power to make changes in the physical and social environment affecting physical activity.

### We do not always feel like we have a say

Participants described a strong sense of belonging and affection for their neighbourhood. In pictures, they showed everyday activities and surroundings, such as nature, playgrounds and architecture. In contrast, they described a negative narrative of their neighbourhood among non-residents and the authorities and feeling unheard when trying to influence their living conditions. They described a discrepancy in the perceptions of the neighbourhood between themselves and the ones in power, including needs and priorities in terms of physical activity, and they did not feel listened to even in dialogue meetings with authorities.

The interview participants expressed frustration with the perception among non-residents that their entire neighbourhood was unsafe, due to the actions of a small group of criminal youths. Despite the neighbourhood having enjoyed a prolonged period without incidents related to gang violence, two negative events occurred during the data collection period. These incidents evoked feelings of sadness, as they appeared to fuel the negative stereotypes projected onto the neighbourhood. Participants also described a sense of collective punishment for this, now and before, as public places and facilities for physical activity appeared to be inadequately maintained compared with neighbouring areas. One participant felt frustrated about the lack of maintenance at the local bus stop:

It used to be messier here than it is now, I think. Just look at the bus stop. It is not even covered in glass or anything, it’s a net that blows straight through. The snow comes through and you’re just like aaaah, it rains on you […] the bench is completely wet or snowy. No one can sit there. I guess it must have been destroyed several times so they have done this instead. – P6 (Female, 35 years old, 1-year resident of the neighbourhood).

The municipality emerged as a prominent actor with priorities opposed to those of the residents in relation to the types of physical spaces and conditions needed to be physically active. Throughout the interviews, participants frequently used terms such as ‘us’ and ‘them’ to distinguish between residents and the municipality:

There is no need to feel sorry for us in that way. No, I know the municipality wrote it in some kind of paper, that they thought this tram would give us better connections. I feel like, don’t they already know that we have lots of buses running frequently, and also, what are we missing? Why would we want to go to town so much? What are we missing then? What are they thinking. – P4 (Female, 53 years old, 9-year resident of the neighbourhood).

**Figure 1:**
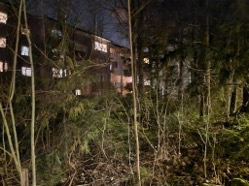
Photo of the woods to depict the closeness to nature (**P9**).

Nature was highly valued among participants as a resource for physical activity, contrasting with the negative reputation of the residential area. Pictures of the closeness of the forest to the residential area were shown (figure 1), and it was highlighted that nature was perceived as an accessible and expense-free option for engaging in physical activity. However, participants expressed a fear that the municipality was destroying ‘their forest’.

Everyone who lives along this street and has the woods alongside like this. They don’t want them to disappear. But they probably will.—P4 (Female, 53 years old, 9-year resident of the neighbourhood).

Despite efforts made to foster dialogue between residents and the municipality, several participants felt as if they were not being listened to, and at the same time they felt pitied. They stressed their efforts to communicate needs and priorities to those with the perceived power, but felt their voices were being disregarded, leading to a sense of neglect as citizens in the neighbourhood. The municipality had held dialogue meetings, seeking input on ways to promote community engagement in the development of the neighbourhood. Some participants perceived these positively while others expressed frustration over how their input did not result in desired outcomes, which P6 expressed felt like a war:

A lot of people have tried to say that like this is crazy [project to build new roads] and the police and ambulance drivers have tried to voice their opinion but they are just running their race like. No, this bicycle path should be this and that and this road like this and everyone is like - but helloo it gets worse. No but it feels like a war. – P6 (Female, 35 years old, 1-year resident of the neighbourhood).

**Figure 2:**
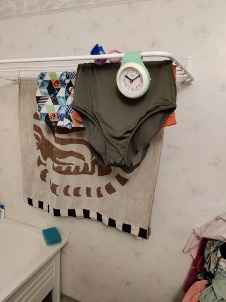
Photo of swimwear that prompted a discussion of women’s perceived safety in the swimming hall (**P6**).

Female participants expressed discomfort exercising in the presence of men and valued the social aspect of being in a room with only women. These participants had previously been regular visitors to the local swimming hall, particularly on Sundays, when there were designated hours exclusively for women (figure 2). When this was discontinued, women such as P9 expressed feeling unsafe when visiting the swimming hall:

When I go to the swimming hall, I ask why they have closed, it was so nice! […] It’s not nice to mix with men and you can’t. You don't feel safe. If it’s with other women, you can do whatever you want.—P9 (Female, 37 years old, 4-year resident of the neighbourhood).

In response to this need for a social and safe space for physical activity, some women had resorted to working out in their homes, using online platforms. Exercising with an online community served as a substitute for meeting in person.

### I need more support but without any pressure

The participants expressed the need for support to be physically active while emphasising the importance of autonomy in choosing how and when to participate in activities. Although they described physical activity as the responsibility of the individual, they expressed hesitancy about their willingness to be physically active.

Participants felt that there were limited opportunities for engaging in physical activity in their residential area. P10 and P11, who had been living in the neighbourhood for over 40 years, expressed frustration at how the opportunities to be physically active had been taken away from them, explaining that there had previously been more organised activities in their neighbourhood, such as buses transporting residents to parks, in the 1990s and early 2000s.

All participants underscored the importance of social and inclusive activities to promote physical activity. Loneliness was recognised as a significant barrier, leading some participants to see going for a walk as useless unless they were seen by others as doing an activity. Belonging to a group and feeling seen by others helped to motivate participants to engage in activities:

Now when I had been sick for two weeks, I went back [to a dance course], and this person said hi, I haven’t seen you for two weeks. How is everything? And wow, someone noticed that I hadn’t been there. But if I skip going for a walk, biking or something? Then no one would notice. So for me it’s better with a bit of pressure.—P3 (Female, 53 years old, 1-year resident of the neighbourhood).

However, some expressed that organised activities that required signing up could be perceived as demanding or an obligation to comply. Being obligated to show up was perceived to diminish their sense of autonomy. They believed that activities should be enjoyable, inclusive and accessible.

Although the participants attributed part of the responsibility for their lack of physical activity to others in their community, they primarily seemed to foster a sense of self-blame. Lack of time, financial constraints and fatigue were highlighted as the main barriers to engagement in physical activity.

Many… many of my friends they don’t want to [exercise] they are lazy you could say [laughs]. They are lazy, I used to be like them, working, too lazy to go to the gym and such.—P9 (Female, 37 years old, 4-year resident of the neighbourhood).

**Figure 3:**
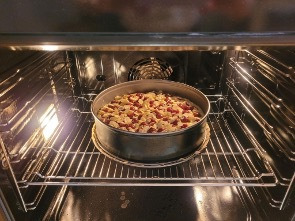
Photo of a pie to symbolise a ‘lazy day’ indoors (**P3**).

Participants also described a perceived expectation to be physically active, that some perceived as a pressure. Some, like P3, expressed a conflict between the lack of willingness and perceived pressure to be more physically active (figure 3):

I’ve heard that, I’ve just heard that there are people who feel like they need to exercise, so during the pandemic in my country you couldn’t do anything. They felt, yes, they became crazy just because they were not allowed to move or run. But not me, I can stay at home for a month and lie on the couch. I don’t feel that.—P3 (Female, 53 years old 1-year resident of the neighbourhood).

## Discussion

In this study, we found that residents living in a socioeconomically disadvantaged neighbourhood in Region Uppsala, Sweden, experienced conflicting values and priorities between themselves and the local authorities regarding physical activity. They described divergent priorities in the development of physical spaces in the neighbourhood and opportunities for physical activity. Four main findings emerged as key sources of tension. First, participants held the belief that being heard equated to having influence, resulting in frustration when their opinions were not reflected in the outcome. Second, a cultural perception regarding the preference for gender-separated exercise spaces conflicted with the Swedish norm of mixed-gender spaces, which aligns with previous research.^17 19^ Third, there was a perceived societal pressure to be active, which contrasted with their willingness and motivation to engage in physical activity. This finding has been demonstrated in prior research.^18^ Lastly, although physical activity was seen as an individual responsibility by the participants, they highlighted the need for societal support to engage in it, which reflects the findings of previous research. Participants consequently expressed frustration at the perceived lack of societal support. The findings of this study shed light on a complex interplay of different values, priorities, perceptions and barriers that influence physical activity behaviours in a socioeconomically disadvantaged neighbourhood in Sweden.

In this study, the participants explained that their attempts to voice their opinions to the perceived authority were often unsuccessful, with the authority acting in accordance with its own values rather than considering those of the residents. We also found a tension between the perception of physical activity being an individualised responsibility and the expressed need for societal support to engage in physical activity. The discourse on physical activity as being the responsibility of the individual has been highlighted in previous literature.^19^ In our study, individualised health responsibility seemed to be hindered by the discrepancy in priorities between residents and the authority, and dialogue meetings in which the residents did not feel listened to. This discourse contrasts with the literature on the environmental determinants of health, which highlights the importance of creating conducive societal environments for physical activity and health.^36^ In Sweden, there is an ongoing debate about the accommodation of gender separation in exercise spaces. This reflects a societal conversation about inclusivity and diversity, raising questions about the balance between individual and cultural preferences and the promotion of equal opportunities in public spaces.

The findings of this study can be viewed through the lens of a framework of soft power and symbolic violence, highlighting the potential limitations of top-down public health interventions in similar settings.^11^ A collision between the values and priorities forced on the citizens by the authorities is described by Mikkelsen *et al*^11^ as ‘symbolic violence’. ‘Soft power’ implies that groups in society take on health behaviours due to a normative power of social values. This study reveals that residents’ values and priorities, influenced by the ‘soft power’ in the local community, differ from the values and priorities of those in power. As described in the framework, the conflicting understanding of what is ‘right’ and ‘wrong’ health behaviour, creates a discrepancy between health knowledge and health behaviours among the residents and may lead to populations resisting public health initiatives.^11^ This can contribute to the low acceptability of health interventions. Previous findings have demonstrated that recommended health behaviours may be perceived as out of reach and that perceived pressure from society might be interpreted as being coercive.^19 20^) Interpreting the results of our study through the lens of Mikkelsen’s framework, a top-down public health intervention, which disregards the values and priorities of the local residents, would yield limited results, as it could be perceived as unattainable by the target population.

The findings of this study underscore the significance of acknowledging the power dynamics and value conflicts inherent in participants’ perceived use of public spaces for physical activity when developing public health interventions. This study emphasises the need for context-specific, participatory approaches that address the complexities in the individuals’ perceptions of physical activity and health. The study’s findings may be applied to similar neighbourhoods with comparable populations in other parts of Sweden and in other European countries. Future research should aim to explore possible differences in perceptions across different risk groups and find ways to bridge the gap in understanding the needs of the specific population for which public health interventions are planned or rolled out.

To the best of our knowledge, no previous study has applied the photo-elicitation methodology to understand the values and priorities associated with physical activity among residents in socioeconomically disadvantaged neighbourhoods in Region Uppsala, Sweden. The use of this methodology is one of the strengths of this study since it facilitated a deeper understanding of participants’ lived experiences.^29 30^ This approach encouraged discussions that revealed the meanings attached to specific places, activities and facilities. The diverse participant demographics, including age, gender and country of birth, added depth to the study’s findings. However, the limited representation of individuals with a high risk of T2D should be acknowledged as a limitation. Furthermore, since the first author, who conducted the interviews, only spoke Swedish, individuals without proficiency in the language were excluded, which may have introduced selection bias. However, it is noteworthy that the native Swedes in this study did not differ significantly from the participants born abroad, in terms of values and priorities, which underscores the complexity of a superdiverse community where language proficiency is only one aspect of the broader diversity that exists. Moreover, there were no clear distinctions in results based on gender or country of birth, except for the preference among women for gender-separated exercise spaces. Further, the authors brought diverse perspectives into the design of the study and analysis and collaborated for over a year in an iterative process, mitigating potential biases. Since the first author had lived in Uppsala before, there was a risk of reproducing stereotypes of the neighbourhood in which this study was conducted. However, by being present in the community, the first author gained a deeper understanding of how local residents used the environment, minimising the risk of preconceptions. In discussion with the others, insider perspectives could be counterbalanced with outsider perspectives to reduce potential biases.

## Conclusion

This study highlights several values and priorities related to physical activity among residents living in a socioeconomically disadvantaged neighbourhood in Uppsala, Sweden. These include a perception that being heard is synonymous with having influence, a preference for gender-separated exercise spaces, a perceived societal pressure to be active and a belief that physical activity is an individual responsibility. Residents’ needs and priorities with regard to physical activity conflicted with those of the authorities. This contributed to mistrust and a feeling of ‘us against them’ and thereby consolidated an individualised discourse of health responsibility among the residents.

Understanding the needs and priorities of the local population is essential when designing public health interventions targeting physical activity in disadvantaged settings. Furthermore, acknowledging and navigating the complex interplay of priorities, values, and power dynamics in the specific context is crucial for their successful implementation.

### AI use statement

This study partly utilized OpenAI’s GPT-4 technology to enhance the manuscript's language quality. Specifically, we employed GPT-4 to suggest synonyms and clarify sentences, thereby improving the overall clarity and readability of the paper.

## supplementary material

10.1136/bmjopen-2024-085356 online supplemental file 1

10.1136/bmjopen-2024-085356 online supplemental file 2

## Data Availability

Data are available on reasonable request. No data are available.
