# Supplementary material for: ‘I’ve just heard that there are people who feel like they need to exercise’: a photo-elicitation study of values and priorities influencing physical activity in a socioeconomically disadvantaged neighbourhood in Uppsala, Sweden
Source: BMJ Open. 2024 Aug 29;14(8):e085356. doi: 10.1136/bmjopen-2024-085356 (PMC11367300; doi:10.1136/bmjopen-2024-085356)
Supplement: online supplemental file 2 [file bmjopen-14-8-s002.pdf]

Interview guide

"I've just heard that there are people who feel like they need to exercise": A photo-elicitation study of values and priorities influencing physical activity in a socioeconomically disadvantaged area in Uppsala Region, Sweden

Please note that this interview guide has been translated from Swedish, and the name of the neighbourhood has been replaced to only using the term "neighbourhood" to avoid stigmatisation.

1. How long have you lived in this neighbourhood and what do you think about it?
   - Have you lived in other areas in Uppsala?

2. Tell me about your neighbourhood. What places do you like?
   - Where in your neighbourhood do you spend time being physically active the most/least?

3. Tell me about the photographs you have chosen. What is in the picture?
   - What would you like to say about this picture?
   - Who uses this place?
   - How do people use this place and what do they usually do there?
   - When are people usually here? Is there any time of the day when people are not here?

<u>Repeat for every picture, omit follow-up questions that have already been covered.</u>

4. What do you think these pictures say about your neighbourhood?
   - Are there other similar places/activities in the area?
   - What do you believe non-residents think about your neighbourhood?

5. Are there any places that makes it easy/difficult to be physically active in your neighbourhood?
   - Are there any places where you do not want to spend time?
   - How do those places look like?
   - How does those places affect you who live here?

6. How would you like your neighbourhood to look like – in terms of places and activities for physical activity?
   - What do you need more of to be physically active/feel good?
   - Would you like to be more physically active? If yes, what would help you?

- Do you think your neighbourhood differs from other neighbourhoods in Uppsala, in terms of possibilities for physical activity?

7. Who decides or has the power in your neighbourhood?
   - What do you think about your own opportunities to influence your neighbourhood?
   - Who do you think is responsible for the well-being of the residents here?
   - How has your neighbourhood changed over the time you have lived here?

- Now we have talked about your neighbourhood and what places and activities that are available for being physically active. Is there anything else you would like to share?
