# Supplementary material for: ‘I’ve just heard that there are people who feel like they need to exercise’: a photo-elicitation study of values and priorities influencing physical activity in a socioeconomically disadvantaged neighbourhood in Uppsala, Sweden
Source: BMJ Open. 2024 Aug 29;14(8):e085356. doi: 10.1136/bmjopen-2024-085356 (PMC11367300; doi:10.1136/bmjopen-2024-085356)
Supplement: Supplementary file 3 [file bmjopen-2024-085356.reviewer_comments.pdf]

PEER REVIEW HISTORY

BMJ Open publishes all reviews undertaken for accepted manuscripts. Reviewers are asked to complete a checklist review form and are provided with free text boxes to elaborate on their assessment. These free text comments are reproduced below.

ARTICLE DETAILS

**TITLE (PROVISIONAL)**	"I've just heard that there are people who feel like they need to exercise": a photo-elicitation study of values and priorities influencing physical activity in a socioeconomically disadvantaged neighbourhood in Uppsala, Sweden
**AUTHORS**	Engelbrektsson, Louise; Ottesen, Hedda; Daivadanam, Meena; Matta, Laran; Alvesson, Helle

VERSION 1 - REVIEW

**REVIEWER NAME**	***Lagstrom, Hanna***
**REVIEWER AFFILIATION**	University of Turku
**REVIEWER CONFLICT OF INTEREST**	Na
**DATE REVIEW RETURNED**	26-Mar-2024

**GENERAL COMMENTS**	First, I would like to thank the authors for interesting manuscript. I have only some comments:<br>1. Abstract: Is it possible to describe more specifically criteria for socioeconomically disadvantage living area(s)?<br>2. It is not always clear what is meant by the word area (compared to references/title). Always living area?<br>3. Discussion is well written, but how about the generalizability of the results, even in other countries?

**REVIEWER NAME**	***Ramji, Rathi***
**REVIEWER AFFILIATION**	Malmö Universitet, Department of Care Science
**REVIEWER CONFLICT OF INTEREST**	Na
**DATE REVIEW RETURNED**	30-Mar-2024

GENERAL COMMENTS	This study describes the perceived norms and values related to physical activity among populations living in disadvantaged neighbourhoods. The study is based on a participatory approach or rather PPI through use of photo elicitation method and provides interesting results regarding the needs of populations living in disadvantaged areas. One major aspect that is lacking is a proper rational as to what the current study adds to what is already known regarding barriers to physical activity among populations in disadvantaged neighbourhoods. There are extensive studies both fro, Stockholm region and the South of Sweden where the most socially vulnerable areas as defined by the Swedish Police Authorities are located. There do exists large Community-based participatory research programs focusing on similar groups where the populations have actively been in identifying needs and co-designing physical activity interventions. The authors neither acknowledge the presence of such studies nor discuss the rational for their own study more clearly.<br><br>In the abstract the authors state that there is little known regarding perceptions about to physical activity about the social environment among disadvantaged populations which says that the authors have not looked into prior literature both from Sweden and elsewhere in the world since they fail to quote any such studies throughout their manuscript. I recommend that the authors do another review of literature to see if studies in similar context in Sweden exist and include them.<br><br>Background<br>In the abstract and background the authors focus on socioeconomically disadvantaged areas, however, do not clearly define this term from a Swedish context point of view..<br><br>Similarly when the authors discuss social environment there is a lack of clarity regarding what it includes since the authors only discuss norms and values in this regard. Do they mean that only the norms and values are part of their social environment? How about social support which later the authors highlight as a facilitator? How about the immediate physical environment including trust in the public system such as health care are they not part of their social environment?<br>Lin 38-39 the authors state certain social groups, but fail to discuss who they are? In my understanding normative interventions often target the majority population rather than certain social groups. This needs to be clarified further.<br><br>Line 45-46 The authors discuss barriers and facilitators in disadvantaged areas.. How can lack of time be a barrier for disadvantaged areas? It must be for the populations living in these areas. Yet again not defining this population clearly provokes further questions such as why lack of time? Secondly, when speaking about weather conditions how does the weather differ in disadvantaged areas compared to more affluent areas? Why is the weather perceived differently in these areas? Does it have to do with the origins of populations living in these areas? Here again, I would like to point out these aspects may seem clearer if the authors define who these people are in a better way?<br><br>The authors also discuss the presence of strong leaders as a facilitator it could benefit for more clarilty if is at a community level or at a national level.<br><br>Methods<br>The authors clearly outline the methods used in this study under the method section. However, the manuscript will benefit from a review of terminology used in the method section since the authors discuss power balance but use words such as*instructed*

**REVIEWER NAME**	***Carroll, Suzanne***
**REVIEWER AFFILIATION**	University of Canberra, Centre for Research and Action in Public Health, Health Research Institute
**REVIEWER CONFLICT OF INTEREST**	Na
**DATE REVIEW RETURNED**	15-Apr-2024

**GENERAL COMMENTS**	This paper applies a photo-elicitation approach in order to understand the norms and values of physical activity of individuals living in a socioeconomically disadvantaged area. Thank you for the opportunity to review this paper.<br><br>Overall, this is an interesting paper but lacking in deep engagement with the breadth of various health behaviour and norms theories. The approach, specifically focusing on soft power and symbolic violence, is not clearly justified. To me, the results do not directly answer the proposed objective of the study – which may be due to a lack of clear definitions in particular regarding the term 'norms'. However, the resultant themes do not all seem to clearly relate to physical activity norms and values of the individuals but appear more intervention co-design and relations between government and community focused. I do not believe the resultant identified four norms as described in the discussion actually meet the definition of norms. Rewriting to bring the links out in a more obvious way is needed. Currently the contribution of this paper to the literature is not clear, though could potentially be made so with some reworking.<br><br>Specific comments:<br>Uppsala Region or Region Uppsala – please be consistent – this currently seems to switch. Uppsala Region seems best to me, but I'm not familiar with the local context/naming convention for areas.<br><br>Abstract:<br>Given the objective of the study focuses on physical activity – I suggest removing the reference to diet as it does not align with the study objective and just creates confusion re study focus.<br>There is some difference between results presented in the abstract and main results section. The abstract is clear, presenting only the 4 subthemes, using different words to those in the results.<br>I'm not convinced the norms framing bears out in the results themes. It's not clearly needed within the framing of the paper as the results don't link well to the stated norms focus and related theory.<br>The abstract conclusion does not clearly relate to the study findings.<br><br>Background:<br>The frameworks/conceptualisation of this research is unclear within the introduction. I think primarily more is needed re the conceptualisation of norms and related theories, and this needs to be presented earlier in the introduction.<br>The term 'norm' has been widely used in different social/behavioural theories often with different definitions – and has been criticised within research for ambiguities caused by such. When you first use the term 'norm' i.e., paragraph 2 please provide a clear definition and relevant references to reduce any ambiguity. There is a great deal of research and theory relating to norms, with great breadth and depth, much of this is not reflected in this introduction though the stated objective is to understand the norms and values of PA for your participants. Greater engagement with the breadth of norms literature is recommended and should be included in the introduction.<br>It is unclear to me what you mean by 'public health interventions' taking a 'normative approach'… and 'evaluated on the norms…'. This may be clarified by provision of a clear definition of 'norms', but I think likely will also needs reworking to ensure clarity of what you mean.<br>What is a 'normative public health intervention'? Please define – and provide an example.<br>When you say 'benefiting certain social groups' – please explicitly state which or provide an example rather than having the reader infer..<br>It is not clear to me why you adopted a framework focused on power dynamics including soft power and symbolic violence. This is an interesting approach though the justification for its use well presented, in particular in how it aligns with your stated research

VERSION 1 – AUTHOR RESPONSE

Reviewe r 1	Abstract: Is it possible to describe more specifically criteria for socioeconomically disadvantage living area(s)?	Thank you for your suggestion. We have revised the abstract to include more specific criteria for defining socioeconomically disadvantaged neighbourhoods.	Page 1-2; line 27-30
	It is not always clear what is meant by the word area (compared to references/title). Always living area?	We agree that this may have been unclear and have now clarified by using "neighbourhood" where appropriate.	
	Discussion is well written, but how about the generalizability of the results, even in other countries?	Thank you for your suggestion. We have added a paragraph of the transferability of the findings at the end of the discussion.	Page 20; line 445-447
Reviewe r 2	This study describes the perceived norms and values related to physical activity among populations living in disadvantaged neighbourhoods. The study is based on a participatory approach or rather PPI through use of photo elicitation method and provides interesting results regarding the needs of populations living in disadvantaged areas. One major aspect that is lacking is a proper rationale as to what the current study adds to what is already known regarding barriers to physical activity among populations in disadvantaged neighbourhoods. There are extensive studies both fro, Stockholm region and the South of Sweden where the most socially vulnerable areas as defined by the Swedish Police Authorities are located. There do exists large Community-based	Thank you for your thorough comment. We acknowledge the importance of clarifying the population in our setting. Many of the studies conducted in socioeconomically disadvantaged areas focus on single ethnic groups and not super diverse populations as is characteristic of whole neighbourhoods in Swedish suburbs, similar to our study areas. We have clarified the challenges that areas characterised by super diversity are faced with, and clarified why there is a need to conduct this type of study focusing on whole populations within specific settings.<br><br>Additionally, we have expanded our literature review to include studies using community-based or co-design approaches and incorporated these references into our manuscript. Furthermore, we have written a paragraph discussing the significance of these methods. We appreciate your input and believe these enhancements strengthen the overall quality and relevance of our study.	Page 3-4; line 68-78<br><br>Page 4; line 87-94

	participatory research programs focusing on similar groups where the populations have actively been in identifying needs and co-designing physical activity interventions. The authors neither acknowledge the presence of such studies nor discuss the rational for their own study more clearly.		
	In the abstract the authors state that there is little known regarding perceptions about to physical activity about the social environment among disadvantaged populations which says that the authors have not looked into prior literature both from Sweden and elsewhere in the world since they fail to quote any such studies throughout their manuscript. I recommend that the authors do another review of literature to see if studies in similar context in Sweden exist and include them.	Thank you for your input. To clarify our focus, we have refined the introduction and research question sections. We emphasise the perspective of priorities and values, as these are crucial for behaviour change and provide deeper insights than mere perceptions. This focus will help us tailor a public health intervention based on the study's results.<br><br>To further clarify the study's focus, we have removed the sentence in the abstract and expanded the introduction section to provide more context of what is already known.<br>As mentioned before, many of the studies that have been conducted focus on single ethnic groups.	Page 3-5
	Background<br>In the abstract and background the authors focus on socioeconomically disadvantaged areas, however, do not clearly define this term from a Swedish context point of view.<br><br>Similarly when the authors discuss social environment there is a lack of clarity regarding what it includes since the authors only discuss norms and values in this regard. Do they mean that only the norms and values are part of their social environment? How about social support which later the authors highlight as a facilitator? How about the	Thank you for these suggestions. We have now defined socioeconomically disadvantaged areas from a Swedish context and outlined the implications they may entail. Additionally, we have clarified the challenges associated with implementing effective public health interventions in such contexts.<br><br>As you mentioned, the social environment encompasses numerous factors that influence physical activity. Further, we have clarified our focus to prioritise understanding the priorities and values associated with physical activity.<br><br>In addition, as the first author was often present in the community, she was able to observe the use of physical spaces, thus enabling the use of this lens in the data analysis. These observations were valuable to further understand the complexity of the social environment.	Page 3-5; line 65-78, 100-103

	immediate physical environment including trust in the public system such as health care are they not part of their social environment?		
	Lin 38-39 the authors state certain social groups, but fail to discuss who they are? In my understanding normative interventions often target the majority population rather than certain social groups. This needs to be clarified further.	We have now clarified that public health interventions are often designed and implemented based on the needs of the general or majority population. We have also clarified the challenges of reaching socioeconomically disadvantaged groups, especially since these are heterogenous with many different needs.	Page 3; line 65-68
	Line 45-46 The authors discuss barriers and facilitators in disadvantaged areas.. How can lack of time be a barrier for disadvantaged areas? It must be for the populations living in these areas. Yet again not defining this population clearly provokes further questions such as why lack of time? Secondly, when speaking about weather conditions how does the weather differ in disadvantaged areas compared to more affluent areas? Why is the weather perceived differently in these areas? Does it have to do with the origins of populations living in these areas? Here again, I would like to point out these aspects may seem clearer if the authors define who these people are in a better way?	We have defined the population more clearly and highlighted the implications of the super diversity in the community. Regarding the barrier of lack of time, we have included references on studies related to other socioeconomically disadvantaged areas as it is relevant to our study context. As these residents often tend to have demanding occupations or physical jobs, it may lead to not having the time to engage in physical activities. Additionally, observations made by the first author in the area revealed that many residents experience stress due to feelings of being an outsider, which we have also incorporated into the background section where we describe the population characteristics.<br><br>Regarding weather conditions, while the weather itself may not differ, it is perceived by individuals from their respective frames of reference. Individuals from different cultures and warmer countries may therefore experience varying levels of comfort engaging in physical activity depending on their perceptions of the weather.	Page 4-5; line 96-104 we also refer to the clarification on page 3; line 68-78
	The authors also discuss the presence of strong leaders as a facilitator it could benefit for more clarilty if is at a community level or at a national level.	We have clarified that the facilitation of presence of strong leaders is on a community level.	Page 5; line 100
	Methods<br>The authors clearly outline the methods used in this study under the method section. However, the manuscript will benefit from a review of terminology	We have reviewed the terminology used in the method section and recognize the importance of using appropriate language. Thank you for this suggestion.	Page 5-10

	used in the method section since the authors discuss power balance but use words such as*instructed*		
	Secondly it is stated that the first author has been engaged with the community ahead of the study which could be regarded as trust building phase, however it is unclear where such interactions had taken place as the context of study does not clarify where in the neighbourhood the author met and socialised with participants. What role exactly did the different gatekepers have in the community? How was the power dynamics in this community? The readers would benefit from knowing more on this so as to understand how participatory the entire process was.	We have clarified how the process went and where the interactions took place.<br><br>Regarding the power dynamics, the first author noted that the stakeholders had a key function in the area as they were often residents themselves and/or worked closely with the community. They had valuable insights of the population and its diversity. Therefore, they were able to help us, both in the recruitment and in the workshops for the larger project.	Page 7; line 152-162<br>Page 10; line 230-233
	It is unclear who the local stake holders were who engaged in the member checking. If the stakeholders were other member than the participants and if the participants themselves were not involved in this process in what way can this be considered as participatory or co-design in what way is this study unique from any other exploration among populations in similar areas. What was the rational to use other stake holders in place of the participants themselves.	After discussions in the research group, we agreed on a better description of the validity checking process, and we have updated the text accordingly.<br><br>This study is not co-designed in itself, but informs the co-design of a public health intervention in the setting, which we have now clarified. Thank you for bringing this to our attention.	Page 7; line 154-156<br>Page 10; line 230-233
	Also in the table it should be Country of Birth not Region. Region means a particular state or place within a country.	Thank you. We have revised the table and are now using “Country of Birth”.	Page 8; Table 1

	Another query regarding the table is the 7 persons born in Sweden and 2 in Europe. It is unclear what this information adds since it is unclear if they were children of Swedish parents or if they may have migrated from another country, If they were native Swedes did they have similar norms and beliefs compared to the others ?	Thank you for this important comment. We have included a discussion of a potential selection bias in the discussion. The native Swedes share similar characteristics as others in the neighbourhoods and did not differ in terms of priorities and values of physical activity. The clarification of the super diversity in this area, where language proficiency is only one of many factors, aims to clarify this, particularly as native Swedes in this sample often faced challenges such as sick leave or lower income, which may have contributed to their lower levels of physical activity.	Page 8; Table 1<br>Page 21; line 460-467
	What do the authors mean by sufficient information power when discussing when the interviews ended.	We refer to the concept of information power by Malterud et al (https://pubmed.ncbi.nlm.nih.gov/26613970/), which was used to assess data adequacy i.e., meaning we reached a point where additional interviews were unlikely to yield substantially new insights or information. We have clarified this in the manuscript.	Page 10; line 213
	If all interviews were conducted in Swedish and half of the population included in this study spoke other languages it seems like the study has in some sense excluded those who could not speak Swedish language. So the question than is if the 15 selected are in some sense the more educated and affluent ones? Such a discussion must also be included in the limitations.	Please see the answer to your comment above.	Page 21; line 461-467
	Results<br>Interesting results, however can be shortened and the citations can include more information particularly in relation to gender and even country of birth, There is a lack of discussion in this regards either.	The results have been slightly condensed, and the citations now incorporate details regarding gender, age, and years resided in the neighbourhood. Additionally, we have clarified the rationale for using years resided in the neighbourhood as a proxy. Participants often described moving to the area as a "breaking point" following a life event that changed their financial situation.<br><br>Further, a paragraph has been added on the diversity of the participants. We did not find notable differences stratified by country of birth and gender, which is already included in the text.	The results section, page: 11-18<br><br>The rationale of using “years resided in the neighbourhood” at page 11; line 245-247<br>Discussion section, page 21; line 465-467

Reviewer 3	This paper applies a photo-elicitation approach in order to understand the norms and values of physical activity of individuals living in a socioeconomically disadvantaged area. Thank you for the opportunity to review this paper.<br><br>Overall, this is an interesting paper but lacking in deep engagement with the breadth of various health behaviour and norms theories. The approach, specifically focusing on soft power and symbolic violence, is not clearly justified. To me, the results do not directly answer the proposed objective of the study – which may be due to a lack of clear definitions in particular regarding the term 'norms'. However, the resultant themes do not all seem to clearly relate to physical activity norms and values of the individuals but appear more intervention co-design and relations between government and community focused. I do not believe the resultant identified four norms as described in the discussion actually meet the definition of norms. Rewriting to bring the links out in a more obvious way is needed. Currently the contribution of this paper to the literature is not clear, though could potentially be made so with some reworking.	Thank you for this thorough feedback. We have carefully reviewed our data with your comments in mind and have come to the conclusion that the terminology " values and priorities" better encapsulates the essence of our findings. As a result, we have revised both the research question and the introduction section to align with this slightly shifted focus. We believe that this adjustment more accurately reflects the nature of our study and better clarifies the significance of the findings within the context.	Introduction, page 3-5<br><br>Research question, page 5; line 106-109
	Specific comments:<br>Uppsala Region or Region Uppsala – please be consistent – this currently seems to switch. Uppsala Region seems best to me, but I'm not familiar with the local context/naming convention for areas.	Thank you for bringing this to our attention. We have revised it to " Region Uppsala" as it is based on the naming conventions in Swedish.	
	Abstract:<br>Given the objective of the	Thank you for bringing this to our attention. We have removed the reference to diet.	Page 1-2; line 27-44

	study focuses on physical activity – I suggest removing the reference to diet as it does not align with the study objective and just creates confusion re study focus.<br>There is some difference between results presented in the abstract and main results section. The abstract is clear, presenting only the 4 subthemes, using different words to those in the results.<br>I'm not convinced the norms framing bears out in the results themes. It's not clearly needed within the framing of the paper as the results don't link well to the stated norms focus and related theory.<br>The abstract conclusion does not clearly relate to the study findings.	We have revised the abstract conclusion to better capture the study's findings.<br><br>We emphasise the perspective of priorities and values, as these are crucial for behaviour change and provide deeper insights than mere perceptions. This focus will help us tailor a public health intervention based on the study's results.<br><br>We have focused on our study's main findings in the abstract, rather than summarise the whole results which might have contributed to the perceived differences. We have double checked that the major findings outlined in the beginning of the discussion and those in the abstract are the same.	
	Background:<br>The frameworks/conceptualisation of this research is unclear within the introduction. I think primarily more is needed re the conceptualisation of norms and related theories, and this needs to be presented earlier in the introduction. The term 'norm' has been widely used in different social/behavioural theories often with different definitions – and has been criticised within research for ambiguities caused by such. When you first use the term 'norm' i.e., paragraph 2 please provide a clear definition and relevant references to reduce any ambiguity. There is a great deal of research and theory relating to norms, with great breadth and depth, much of this is not reflected in this introduction though the stated objective is to understand the norms and values of PA for your	Thank you for this comment. As mentioned, we have re-examined our data and made slight adjustments in the terminology, changing from "norms" to "priorities" and "values". We hope that this adds clarity and think it has a better alignment to our results.<br><br>Regarding the framework, we have clarified how it was used in the text. Since the framework was only used to understand the findings of our study, not the design of it, we have chosen to remove it from the Introduction section and instead focus on discussing the results in relation to the framework in the Discussion section, which we believe is more appropriate for our article.	Page 5; line 101-109<br><br>Page 20; line 424-439

	participants. Greater engagement with the breadth of norms literature is recommended and should be included in the introduction.		
	It is unclear to me what you mean by ‘public health interventions’ taking a ‘normative approach’… and ‘evaluated on the norms…’. This may be clarified by provision of a clear definition of ‘norms’, but I think likely will also needs reworking to ensure clarity of what you mean.<br>What is a ‘normative public health intervention’? Please define – and provide an example.<br>When you say ‘benefiting certain social groups’ – please explicitly state which or provide an example rather than having the reader infer..	We have revised the terminology to avoid confusion. With normative public health interventions, we refer to that it is often health authorities that set the recommendations based on the needs of general populations.<br><br>We have also clarified the context of the setting and that these interventions are targeting the whole population rather than groups in socioeconomically disadvantaged settings.	Page 20; line 434-439<br><br>Page 3-4; line 65-78
	It is not clear to me why you adopted a framework focused on power dynamics including soft power and symbolic violence. This is an interesting approach though the justification for its use well presented, in particular in how it aligns with your stated research objective. Perhaps incorporating other definitions / conceptualisation of norms including related theories may help – for example – p4 line 23/24 you state ‘prescribed norms might be perceived as too far out of reach’ – what is a prescribed norm? Consideration of Cialdini’s work could add depth, nuance and clarity. That is – is this a descriptive norm or an injunctive norm? Something else? There is a great deal of depth and complexity around ‘norms’	Thank you for this comment. The framework describes mechanisms behind why top-down or normative public health interventions often fail to succeed in socioeconomically disadvantaged areas. As above mentioned, we have chosen to remove the paragraph on the framework from the Introduction section and instead focus on discussing our findings in relation to the framework in the Discussion section, which we find is more appropriate.<br><br>We view norms as a set of values, where “norms too far out of reach” refer to the recommendations often set by health authorities that sometimes are in conflict with local needs and values. An example could be a recommendation of walking 10,000 steps a day, which may be seen as too far out of reach for someone who face barriers such as having a stressful job, or has migrated from a warmer country abroad to Sweden and may not want to go out walking in the cold temperatures.<br><br>Thank you for the suggestion to incorporate the theories. With the new focus on values and priorities, we find that this is no longer relevant.	Page 20; line 424-439

	which is currently missing from this paper. Also consider various health behaviour theories such as the Theory of Planned Behaviour / Integrated Behaviour Model as well as definitions such as subjective and local norms (e.g., Carrus et al. 2009; Fornara et al. 2011). Currently, the norms focus within this paper is unclear, it does not come across clearly enough, nor is it well integrated throughout the paper (analyses, results, themes developed, and discussion) – to me it needs to be incorporated with more intention, depth and clarity, or removed.	As this study informs the co-design of a public health intervention in the neighbourhood, we see a value of interpreting the results through this framework to understand the underlying mechanisms.	
	Methods:<br>What epistemology underpins this study? What potential biases exist based on researcher background? and what approaches did you use to reduce these?	Thank you for this input. We have clarified our adoption of a social constructivist paradigm and have included a description of our efforts to mitigate potential biases in the Discussion section.	Page 6; line 128-130<br><br>Page 9; line 183-186<br><br>Page 21-22; line 466-473
	Please add detail re the interpretive engagement framework rather than just the reference.	We have clarified that the interpretive engagement framework was utilised to interpret the pictures and interview data holistically, rather than as separate entities. In consideration of various approaches to interpreting photo elicitation data, we found that treating the images and data as an integrated whole provided a suitable comprehensive understanding of the data.	Page 5-6; line 124-128
	P5 line 22 – check your expression – 'higher unhealth rates' – perhaps 'higher rates of poor health' – or something similar	Thank you for your suggestion. We have reviewed the expression and revised it to "higher rates of poor health" for clarity and precision.	Page 6; line 139
	Thank you for the description of the setting – this is a useful inclusion.	Thank you, we are happy to hear it was useful!	
	P5 line 31/32 'first author informed…' sounds odd, perhaps 'provided information'?	Thank you for this suggestion. We have reviewed the expression.	Page 7; line 157

	Line 34/35 – 'engaging in different activities…' – such as what?	We have now included specific examples of the activities in which the first author engaged with the community.	Page 7; line 157-162
	Recruitment – you used purposive sampling – can you provide detail? In what way was it purposive? Were you seeking coverage across a range of sociodemographic variables? Physical activity behaviour? Something else?	We have provided detail of how we sought coverage across socio demographic variables. As this study informs the design of a co-design intervention, we strived to include individuals with demographics similar to the target population of the intervention.	Page 7; line 165-170
	Data analysis:<br>How many researchers were involved in the coding? Was the coding done completely independently then researchers convened to agree on codes…? Other options? Please provide more details	We have provided details of how the coding process was conducted and which authors were involved.	Page 10; line 220-223
	I note that the final stage of analyses involved applying the lens of soft power and symbolic violence – however this does not come across in a meaningful way in your results/discussion. Perhaps it needs to be more explicitly brought out (or removed)? I currently don't see the value in the approach – i.e., you need to be more convincing with it justification and application.	We have moved the theoretical framework to the discussion, as we used it as a lens to interpret the implications of the findings. As previously mentioned, this study informs the co-design of a public health intervention in the neighbourhood. Therefore, we see a value of interpreting the results through this framework to understand the underlying mechanisms.	Page 20; line 424-439
	Results:<br>Subtheme 'we do not always feel like we have something to say' – doesn't reflect the categories/subcategories, it could be a translation issue. Consider rewording this.	Thank you. This was a translation issue, and have revised it to "We do not always feel like we have a say" to better capture how the residents' did not feel heard, as they had different priorities than the authorities.	Page 11; table 2
	I feel the themes/categories etc and their presentation need to be more obviously related back to your research objective physical	Thank you for this comment. We hope that the new terminology of priorities and values have added clarification to the results section, including themes and categories.	Page 11-18

	activity norms and values for individuals. Some seem more focused on issues in relations between government and individuals and not physical activity norms / values per your stated objective. Please edit to make linkages to the objective more obvious.		
	Discussion<br>P22 line 24/25 – what do you mean 'health norms' here?<br>You identify four main norms emergent from your analyses – though these are not presented as such in the results. It is not clear to me that I would define these four concepts as 'norms' – please check how these relate to definitions of norms. For example, the first is an unmet expectation rather than a norm. Each of the others also have their issues in relation to being defined as 'norms'.<br><br>P 13 lines 10-15: "As described in the framework of soft power and symbolic violence by Mikkelsen, the conflict between different norms shaped by soft power and symbolic violence create a discrepancy between health knowledge and health behaviours among the residents and may lead to a counter norm resisting public health initiatives (8)" it's not clear to me how this works or what this looks like, i.e., how the power structures lead to the difference in knowledge and behaviour, or essentially residents not being physically active as a way to resist a public health initiative – can you provide an example to support this	We have revisited our data to find that the words "values and priorities" better describe the scope of our study. Therefore, we present our results as "findings" instead of "norms".<br><br>With health norm, we refer to the recommendations of the public health intervention, for example being physically active 150 minutes per week. We have now clarified this and are now using the word "recommendations".<br><br>If the public health intervention is designed and implemented top-down, disregarding the needs of the local residents, it will most likely yield limited results as the residents may find it unattainable or "not for them". We believe this is an important implication to bring to attention. The participants in this study did not necessarily find it important to be physically active, and with the existing power dynamics and conflicts with the authorities, it is crucial to tailor the design of an intervention to their specific priorities and values. If not, we believe that these participants could resist this public health intervention, as they would find it "forced" upon them and may be designed or have recommendations that are not according to their own needs.	Page 21; line 424-439

	assertion?<br>P13 line 51: “The implications drawn from this study suggest that a normative public health intervention in this setting would yield limited results, as the health norms could be perceived as unattainable.” Whilst this is likely true – I don’t see that it necessarily develops from your findings. When you say ‘normative public health intervention’ do you mean one that recommends 150 minutes of MVPA per week? OR something else – its not clear so add in needed detail – and if so, this doesn’t really link to your objectives or your findings.		

VERSION 2 – REVIEW

**REVIEWER NAME**	***Ramji, Rathi***
**REVIEWER AFFILIATION**	Malmö Universitet, Department of Care Science
**REVIEWER CONFLICT OF INTEREST**	Na
**DATE REVIEW RETURNED**	15-Jul-2024

**GENERAL COMMENTS**	The manuscript has been revised taking into consideration the suggestions and comments provided. Good luck with your further work!

**REVIEWER NAME**	***Carroll, Suzanne***
**REVIEWER AFFILIATION**	University of Canberra, Centre for Research and Action in Public Health, Health Research Institute
**REVIEWER CONFLICT OF INTEREST**	Na
**DATE REVIEW RETURNED**	02-Aug-2024

**GENERAL COMMENTS**	I wish to thank the authors for so thoroughly attending my comments. I believe that this version is much improved.
